# [Expression of Concern] Suppression of SIK1 by miR-141 in human ovarian cancer cell lines and tissues

**DOI:** 10.3892/ijmm.2025.5627

**Published:** 2025-09-04

**Authors:** Jin-Long Chen, Fang Chen, Ting-Ting Zhang, Nai-Fu Liu

Int J Mol Med 37: 1601-1610, 2016; DOI: 10.3892/ijmm.2016.2553

Following the publication of this paper, for the western blots shown in Fig. 1A, where the expression levels of salt-inducible kinase 1 were detected in ovarian cancer tissues, it was drawn to the Editor's attention by a concerned reader that the β-actin loading controls for patients 4, 6 and 7 were strikingly similar in appearance. Moreover, several of the protein bands in this figure were strikingly similar to data which subsequently appeared in another paper written by different authors at different research institutes that was published in the journal *Experimental and Therapeutic Medicine*, although this paper has since been retracted.

The authors were contacted by the Editorial Office to offer an explanation for this apparent anomaly in the presentation of the data in this paper, although up to this time, no response from the authors has been forthcoming. Owing to the fact that the Editorial Office has been made aware of potential issues surrounding the scientific integrity of this paper, we are issuing an Expression of Concern to notify readers of this potential problem while the Editorial Office continues to investigate this matter further.

